# Annotations

**Published:** 1888-06-30

**Authors:** 


### Annotations.

**Provident Special Hospitals.**

The general tide of public opinion seems to be flowing towards the pay system in all hospitals. Dissentients there are, and will be, whose voices must be listened to with respect, and whose objections must be justly weighed, but the great voice—the public, or at least of that important section of the public, the middle class—proud and self-respecting, though struggling hard—utters no uncertain sound. "We are too honest to take as a gift a service we can pay for in some degree: but we cannot afford the high fees asked—and reasonably asked, we admit—by men who have attained special distinction in medicine and surgery. Therefore we are worse off not only than our richer but than our poorer brethren, who get for nothing what we would gladly pay for." Against this is set the grievance of the general practitioner, who says that provident hospitals rob him of his patients or force him to attend them at starvation rates. It does not need more than a cursory visit to the poorer parts of any large town to see that there is a class of practitioners, qualified and registered as duly as the highest in the profession, who, without the competition of provident hospitals, work at starvation rates. "Visit and medicine one shilling," can hardly be regarded as anything better. But if there be a reasonable ground of protest against the pay system being introduced into general hospitals, there can be none in the case of institutions for the treatment of special diseases. A specialist is a man who, besides going through the ordinary medical curriculum, has made special study of one branch of disease, usually a difficult one which the general practitioner, in the multiplicity of the calls on his attention, has not observed closely. Presumably the specialist who has observed it, who devotes his life to observing it, knows more about it than other men; and it is hard on a patient who knows or suspects himself to be suffering from the disease the specialist has made a study of, that he cannot consult one, because hospitals will not take from him payment according to his means, and it is impossible for him to give a fee beyond them. The introduction of the pay system into our hospitals would obviate this difficulty, and prevent the growth of the demoralizing tendency in the middle-class patient to scheme for a gratuitous treatment to which he has no right.

**The Celt and the Teuton.**

In this month's number of the *Universal Review*, Mr. Grant Allen writes a fervid article on "The Revolt of the Celt." The Celtic race, conquered and pushed northward and westward centuries ago by the mailed hand of the Saxon, has not only preserved its marked racial characteristics in these fastnesses, but is now pouring its overplus of population back into the Teutonised eastern and southern districts, effecting a peaceful reconquest, which will nevertheless effect a complete change in the English nation and in English politics. Mr. Grant Allen is an ardent Celtophil; according to his notion, the millennium is to begin when the Celt holds undisputed the reins of power. Whatever we may think of his politics, we must admit that "The Revolt of the Celt" is an obvious fact, and the distinction he draws between the two races he contrasts—the same contrast was vividly depicted long ago by Eugène Sue in his romance, "Le Mystère du Peuple,"—is ethnically true. The Celt has a higher, a more distinctly individual self-respect than the Teuton: he will not yield to constituted authority simply because it is constituted authority; he stands on his rights and obeys only when he so chooses. The writer illustrates his position by describing the most Teutonic and the most Celtic nations on the Continent of Europe, the Germans and the French. "The Germans, intelligent, docile, obedient, heavy, submit to be ruled by the iron hand of the sternest Moltke or Bismarck they can find. . . . The French reject the principle of authority, the divine or hereditary right of one man to interfere with another man's movements; they have born within them the sentiment of liberty and the doctrine of government by the sufficient reason." True; and what was the result nigh upon twenty years ago. A score of disunited states formed themselves into one great strong empire on the one hand, because the Teutons, when they had found their leader, knew how to follow and obey him. On the other, the Celts, who had accepted, though restlessly, as is their nature, a despotism less worthy than any Hohenzollern would enforce, threw it off just when the highest patriotism would have advised patience, and still carries the principle of individualism so far that every change of government threatens a revolution. They cannot learn when it is noble to obey, and though the Celtic race has produced many great men, it usually fails, from its too great individualism, its lack of mutual trust and cohesion, in forming great nations. Without union, permanent strength is impossible, or permanent order without subordination. This parable of the nations holds good of all coummunities, those with which The Hospital deals, among the rest. We need both Celts and Teutons in all places and institutions; but when we have Celtic individualism, Celtic genius in our leaders, let those of us who have not genius show the Teutonic nobility of loyal obedience.

**Certain Knaves and Certain Fools.**

We do not pretend to remember who first made the sage remark, *populus vult decipi*, but it might well have been some sad and sober Roman doctor, who saw the fickle and credulous populace flocking past his door to enter that of some audacious charlatan, Greek slave, or Oriental necromancer, who professed to cure all ailments by esoteric charm and mystic drug. Nothing is more strange, more annoying, or more pitiful than the way in which the public pass by the honest practitioner, who, having studied his art earnestly, will promise no more in the way of cure than his knowledge tells him is assuredly possible, to crowd the consulting-rooms and fill the pockets of the pretentious quack. It proves, indeed, a complete ignorance of the simplest facts of anatomy and pathology that there are still to be found people who believe in the man who professes to eradicate cancer "without the knife," or restore the lost lung-tissue in the last stages of phthisis. Men are a little given to this folly, women are its slaves; and she who would not entrust the making of a cotton gown to an untrained dressmaker, will put her life at the mercy of an ignorant pretender, who has gained his notoriety only by blatant and unscrupulous advertising. We do not wish to make out that medicine is an eleusinian mystery which only the few may grasp; it is a science open to all who have an ordinary amount of brains, and the will and time to study it, and therein lies its strength—that it does not fear investigation. We will own, too, that we have a great respect for many so-called "old wives' cures," which are usually founded on common sense, and very sound traditions. We will even admit that the quacks have sometimes brought about marvellous cures—especially where the disease consisted chiefly of laziness and egoism. These admissions do not invalidate the obvious principle that a man who has been taught his work will do it better than one who has had no training for it. No one, we should think, would deny this principle as a general law, yet experience shows us that a large number of people prefer the uneducated to the educated doctor. A recent trial in Paris brought this out in a marked manner. A certain famous quack of Montmartre was brought up for trial on a charge of practising medicine without a legal qualification. Not at all; he brought out his diploma and showed it to the judge, who then asked why he did not practise in a legitimate way. "Because it would not pay," he explained. He had tried and failed; then he took to commerce, and got into the habit of giving his friends the benefit of his medical studies. He found then that the public who would have none of him as a professed doctor would give the commercial traveller almost any fee he chose to ask, and so he drifted into the position of a prosperous quack. Of course, the law could not touch him, but as he left the court he made one request: "Do not disclose the fact that I am a qualified doctor, or I should lose all my practice."

